# Supplementary material for: Quantum Mechanical Study of Oxygen Ligands Protonation for the Stable States of the Laccase Active Site
Source: Int J Mol Sci. 2023 Feb 3;24(3):2990. doi: 10.3390/ijms24032990 (PMC9917769; doi:10.3390/ijms24032990)
Supplement: Supplementary file 1 [file ijms-24-02990-s001.zip › ijms-2191833-supplementary.pdf]

Quantum Mechanical Study of Oxygen Ligands Protonation for the Stable States of the Laccase Active Site

Sergei Gavryushov,[1,2*] Nikolay N. Kuzmich,[2,3] Konstantin M. Polyakov [1]

[1] Engelhardt Institute of Molecular Biology, Russian Academy of Sciences, Vavilova str. 32, Moscow, 119334, Russian Federation;

[2] Sechenov First Moscow State Medical University, 2-4 Bolshaya Pirogovskaya str., Moscow, 119991, Russian Federation;

[3] Smorodintsev Research Institute of Influenza, WHO National Influenza Centre of Russia, 15/17 Professor Popov str., Saint-Petersburg, 197376, Russian Federation;

[*] Correspondence: sergei_gavryushov@yahoo.com

Supplementary Information

S1. Poisson-Boltzmann calculations

Results for the PB calculations of changes of the mean electrostatic energy at monovalent cation transfers from infinity to the entrance to channels T2 and T3 and the further transfers from the channel entrance to positions of the oxygen ligands W2+2 and W3+2 are summarized in Tables S1 and S2 for the reduced and oxidized states of the TNC, respectively. All data are given as the lower and upper limits of values obtained at varying the poorly determined parameters of the model as described in Section 2.2. They are written in brackets, marking three diapasons of values: $((\Delta E_{\min}, \Delta E_{\max})|_{\varepsilon=10}$,$(\Delta E_{\min}, \Delta E_{\max})|_{\varepsilon=4}$, $(\Delta E_{\min}, \Delta E_{\max})|_{\varepsilon=2})$. The first diapason of energy changes corresponds to the highest estimation of the dielectric constant of volume occupied by protein's atoms (ε=10), the second diapason corresponds to the value ε=4 and the third diapason corresponds to the lowest estimate for protein interior polarization (ε=2).

Table S1. Reduced state of the TNC. PB-calculated changes of the electrostatic energy (kcal/mol) at transfers of a monovalent cation from infinity to positions WX+2 in channel TX (X denotes the channel index). The transfer is divided in two transfers by a position at the channel entrance. The TNC oxygen ligand WX is OH^-. Other oxygen ligands are commented in column "Channel TX". Energy changes are given in diapasons of possible values for each of three different protein dielectric constants.

transfer / channel TX	infinity → entrance	entrance → WX+2	total (infinity → WX+2)
T2 (W3 = H_2O)	((+1,+1),(+1,+1),(+2, +2))	((-2,-1),(-3,-1),(-4,+2))	((-1,0),(-2,0),(-2,+4))
T3 (W2 = H_2O)	((+1,+1),(+2,+4),(+7,+8))	((-5,-2),(-8,-3),(-15,-4))	((-4,-1),(-6,+1),(-8,+4))

Table S2. Oxidized state of the TNC. PB-calculated changes of the electrostatic energy (kcal/mol) at transfers of a monovalent cation from infinity to positions WX+2 in channel TX (X denotes the channel index). The transfer is divided in two transfers by a position at the channel entrance. The TNC oxygen ligand WX is OH^-. Other oxygen ligands are commented in column “Channel TX”. Energy changes are given in diapasons of possible values for each of three different protein dielectric constants.

transfer / channel TX	infinity → entrance	entrance → WX+2	total (infinity → WX+2)
T2 (W1 = OH^-, W3 = H_2O)	((+1,+1),(+1,+2),(+2,+3))	((+1,+2),(+3,+6),(+5,+11))	((+2,+3),(+4,+8),(+7,+14))
T2 (W1 = OH^-, W3 = OH^-)	((+1,+1),(+1,+2),(+1,+3))	((0,+1),(+1,+4),(+2,+8))	((+1,+2),(+2,+6),(+3,+11))
T3 (W1 = O^{2-}, W2 = H_2O, W3+1 = OH^-)	((+1,+1),(+1,+4),(+3,+7))	((-9,-6),(-11,-18),(-18,-30))	((-8,-5),(-10,-14),(-15,-23))
T3 (W1 = O^{2-}, W2 = OH^-, W3+1 = H_2O)	((+1,+1),(+1,+4),(+4,+8))	((-1,+1),(0,+5),(+2,+11))	((0,+2),(+1,+9),(+6,+19))
T3 (W1 = OH^-, W2 = OH^-, W3+1 = H_2O)	((+1,+2),(+2,+4),(+4,+9))	((+1,+3),(+6,+11),(+13,+20))	((+2,+5),(+8,+15),(+17,+29))
T3 (W1 = OH^-, W2 = H_2O, W3+1 = H_2O)	((+1,+2),(+2,+4),(+4,+10))	((+3,+5),(+9,+14),(+18,+27))	((+4,+7),(+11,+18)),(+22,+37))
